# Dynamical systems, attractors, and neural circuits

**DOI:** 10.12688/f1000research.7698.1

**Published:** 2016-05-24

**Authors:** Paul Miller

**Affiliations:** 1 Volen National Center for Complex Systems, Brandeis University, Waltham, Massachusetts, 02454-9110, USA

**Keywords:** hidden Markov model, Point attractors, Marginal states, line attractors, continuous attractors, Oscillating systems, cyclic attractors, limit cycles, strange attractors, Heteroclinics

## Abstract

Biology is the study of dynamical systems. Yet most of us working in biology have limited pedagogical training in the theory of dynamical systems, an unfortunate historical fact that can be remedied for future generations of life scientists. In my particular field of systems neuroscience, neural circuits are rife with nonlinearities at all levels of description, rendering simple methodologies and our own intuition unreliable. Therefore, our ideas are likely to be wrong unless informed by good models. These models should be based on the mathematical theories of dynamical systems since functioning neurons are dynamic—they change their membrane potential and firing rates with time. Thus, selecting the appropriate type of dynamical system upon which to base a model is an important first step in the modeling process. This step all too easily goes awry, in part because there are many frameworks to choose from, in part because the sparsely sampled data can be consistent with a variety of dynamical processes, and in part because each modeler has a preferred modeling approach that is difficult to move away from. This brief review summarizes some of the main dynamical paradigms that can arise in neural circuits, with comments on what they can achieve computationally and what signatures might reveal their presence within empirical data. I provide examples of different dynamical systems using simple circuits of two or three cells, emphasizing that any one connectivity pattern is compatible with multiple, diverse functions.

## Introduction

When we try to understand any biological process, our models of the system matter. Our ideas of how a parameter impacts the system or how a variable responds to manipulations of the system determine what questions we attempt to answer and which experiments we perform. These ideas are based on our own mental models, which can be misleading if not founded on the appropriate dynamical principles ^1, 2^.

In this article, I hope to provide a short introduction to the types of dynamical system that arise in neural circuits. In doing so, I explore why there is a perhaps surprising lack of consensus on the nature of the dynamics within mammalian neural circuits—modelers aiming to explain various cognitive processes commence from seemingly incompatible starting points, such as chaotic systems, oscillators, or sets of point attractor states. I briefly consider hallmarks and support for each paradigm and summarize how—in spite of the tremendous quantity of electrophysiological data—room for debate remains as to which type of dynamical system is best used to model and understand brain function.

## Hidden variables within neural data

Neural circuits are nonlinear dynamical systems that, in principle, can be described by coupled differential equations ^3^. However, the relevant continuous variables necessary for a full description of the behavior of a functioning neural circuit are typically hidden from us. Minimally these include the membrane potential of every cell, but recordings of neural activity in behaving vertebrates such as mammals are limited to a small subset of cells. Even if all recorded cells reside in one circuit that we wish to describe, the circuit, which could be distributed or compact, receives inputs from tens of thousands of other neurons, whose activity is unknown. Moreover, even a continuous variable such as membrane potential is most commonly observed only at the discrete times of voltage spikes. Therefore, our descriptions of neural circuits require us to infer the behavior of underlying hidden variables when we observe only a sparse number of them.

Numerous other properties impact the ongoing behavior of a cell and the circuit as a whole. These may include the number of vesicles of neurotransmitter and their voltage-dependent release probabilities at each synaptic connection or the cell-average states of activation and inactivation of the various ion channels. Calcium concentration and spatial distributions of all these values can also affect neural activity. Such an overwhelming abundance of known unknowns makes a full or complete description impossible and helps explain why we not only fail to have a concrete, detailed explanation of the behavior of most neural circuits but even do not know which dynamical system provides the best model of the behavior.

## Classes of dynamical system

A dynamical system is any system that changes in time and can be described by a set of coupled differential equations. A pendulum is a simple example, the Hodgkin-Huxley model of a neuron a more complicated one, and the coordinated activity of all neurons in a brain an intractable one. To characterize a dynamical system rigorously, one should know how the rate of change of all relevant variables depends on the combination of their instantaneous values. One then can simulate how they change in time from any initial condition and plot these co-varying variables together as a trajectory. If a small change in initial conditions leads to identical behavior after some transient period, then the system possesses a point attractor state—trajectories converge if their initial difference is not too large. If the system is an oscillator, trajectories converge to a particular loop—a limit cycle—in which differences in initial conditions are maintained over time as a fixed phase offset. If trajectories diverge from each other across a broad range of initial conditions while all variables remain bounded, then the system is chaotic.

Here we consider circuits with only two or three neurons to provide examples of many different types of dynamics. In general, a system with hundreds or thousands of neurons—so that we would need a space of hundreds or thousands of dimensions to plot the dynamics as the coordinated set of membrane potentials or firing rates of all neurons—could contain point attractors, limit cycles, and regions of chaos depending on which subsets of cells were more strongly active for one period of time. The richness of such high-dimensional systems and their relevance to cognitive function make them an important area of current study ^4– 10^.

## Point attractors

**Figure 1.**
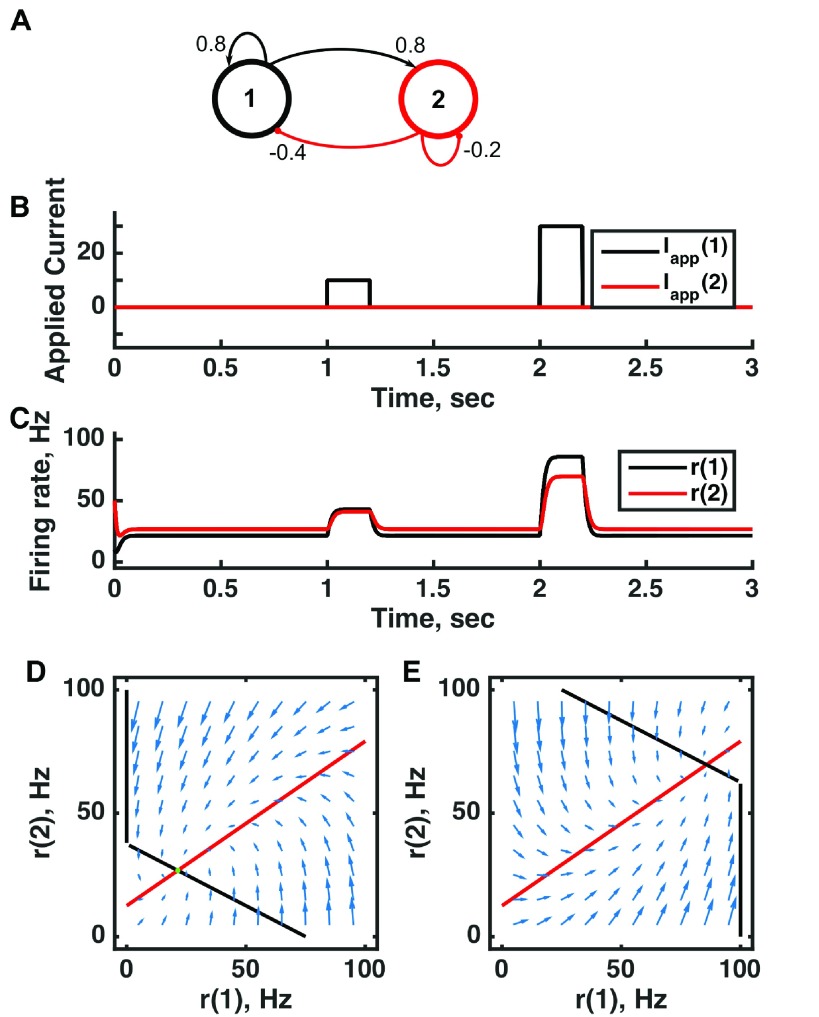
A single point attractor is present without input and a different one with input in a threshold-linear two-unit circuit. ( **A**) Diagram of the model circuit. Arrows indicate excitatory connections, and balls indicate inhibitory connections between units. ( **B**) Applied current as a function of time. Two different sized pulses of current are applied to unit 1. ( **C**) Firing rate as a function of time in the coupled network. During each current step, a new attractor is produced, but following current offset the original activity state is reached. ( **D**) Any particular combination of the firing rates of the two units (x-axis is rate of unit 1, y-axis is rate of unit 2) determines the way those firing rates change in time (arrows). Starting from any pair of firing rates, any trajectory following arrows terminates at the point of intersection of the two lines. Red line: nullcline for unit 2—the value of r(2) at which dr(2)/dt = 0 (its fixed point) given a value of r(1). Since unit 1 excites unit 2, the fixed point for r(2) increases with r(1). Black line: nullcline for unit 1—the value of r(1) at which dr(1)/dt = 0 (its fixed point) given a value of r(2). Since unit 2 inhibits neuron 1, the fixed point for r(1) decreases with r(2). The crossing point of the nullclines is a fixed point of the whole system. The fixed point is stable (so is an attractor state) because arrows converge on the fixed point. ( **E**) As in (D) but the solution during the second pulse of applied current. The applied current shifts the nullcline for r(1) so that the fixed point of the system is at much higher values of r(1) and r(2). For parameters, see supporting Matlab code, “dynamics_two_units.m”.

A point attractor state is equivalent to a stable fixed point of the dynamics, such as the bottom of a bowl with a ball in it. No neural circuit *in vivo* can strictly be in a point attractor state, as that would require all variables (such as membrane potentials) to be static. However, it may be reasonable to consider a broad average across variables, such as the mean firing rate of a large group of neurons, to be stationary following any initial transient response to a fixed input. Simple systems without feedback operate in such point attractor states if cells have one value of firing rate in the absence of stimulus and, typically following a period of adaptation, shift to a different stable firing rate in the presence of a stimulus ( Figure 1). Neurons in the sensory periphery appear to behave in this manner.

Variability in the spiking of a neuron—both within a trial and between trials—appears to be at odds with a point attractor description, which suggests a stable, stationary set of firing rates. However, such variability can be attributed to various noise terms that lead to each neuron’s spikes being produced randomly (say, as a Poisson process) with probability that depends on the rate—a hidden variable, which could be static and deterministic—and/or to noise-driven fluctuations in the rate about its stable fixed point. Thus, a point attractor framework is not incompatible with ever-varying neural activity, especially, as we shall discuss below, for systems with many attractor states.

### Multistability and memory

**Figure 2.**
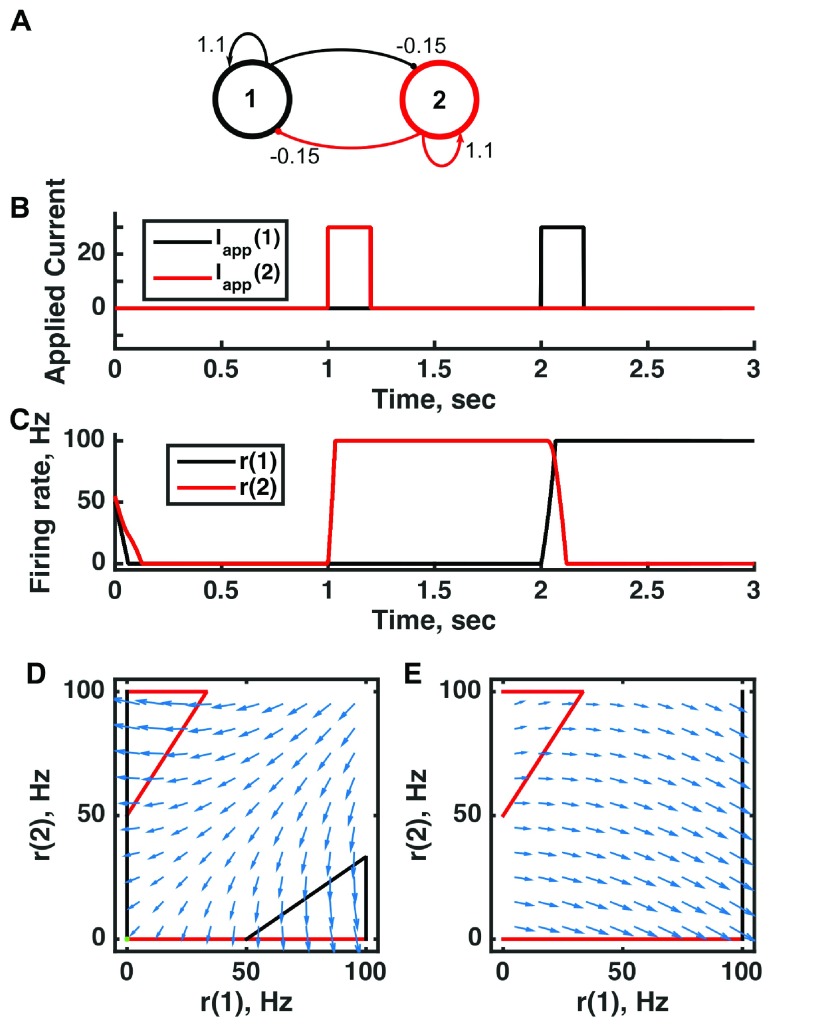
A multistable attractor network can be switched between states to encode distinct memories through persistent activity in a threshold-linear two-unit circuit. ( **A**) Diagram of the model circuit. Arrows indicate excitatory connections, and balls indicate inhibitory connections. The strong self-excitatory feedback renders each unit unstable once active. ( **B**) Applied current as a function of time. The first pulse is applied to unit 1, the second to unit 2. ( **C**) Firing rate as a function of time in the coupled network reveals three different activity states: both units inactive or either one active. The activity persists after offset of the applied current—a signature of multistability—so retains memory of past inputs. ( **D**) Any particular combination of the firing rates of the two neurons (x-axis is rate of neuron 1, y-axis is rate of neuron 2) determines the way those firing rates change in time (arrows). Depending on the initial pair of firing rates, a trajectory following arrows terminates at one of the points of intersection of the two lines, either (0,0) or (100,0) or (0,100). The intersections at the midpoints of the lines are unstable—if activity of unit 1 is under 50 Hz, it decays to 0; if it is over 50 Hz, it will increase to 100 Hz (if unit 2 is inactive). Red line: nullcline for neuron 2—the value of r(2) at which dr(2)/dt = 0 (its fixed point) given a value of r(1). Since neuron 1 excites neuron 2, the fixed point for r(2) increases with r(1). Black line: nullcline for neuron 1—the value of r(1) at which dr(1)/dt = 0 (its fixed point) given a value of r(2). Since neuron 2 inhibits neuron 1, the fixed point for r(1) decreases with r(2). The crossing point of the nullclines is a fixed point of the whole system. The fixed point is stable (so is an attractor state) because arrows converge on the fixed point. ( **E**) As in ( **D**) but the solution during the second pulse of applied current. The applied current shifts the nullcline for r(1) so that the only fixed point of the system is at (100,0). For parameters, see supporting Matlab code, “dynamics_two_units.m”.

When a system possesses multiple point attractor states in the absence of stimuli, then the history of prior stimuli can determine the neural circuit’s current activity state—the particular attractor in which it resides—so the system can retain memories ( Figure 2). In some of the most important pioneering work in computational and theoretical neuroscience ^11– 14^, Grossberg and Hopfield demonstrated how such discrete memory states can form via activity-dependent changes in the strength of connections between coactive neurons during stimulus presentation. While the initial analyses of the capacity (number of memories held) of such networks relied on binary neurons that were either on or off and updated in discrete time steps ^12^, the principle of memory formation and memory retrieval via an imperfect stimulus has been demonstrated in continuous firing rate models in continuous time ^14, 15^ and in circuits of model spiking neurons ^16^. These pattern-learning systems, known generically as autoassociative networks, provide great insight into how memories can be distributed across overlapping sets of cells and retrieved from imperfect stimuli via pattern completion to a point attractor state.

### Inhibition-stabilized network

The value of a dynamical systems approach to understanding neural-circuit behavior that is otherwise highly counter-intuitive—even paradoxical—is exemplified by the inhibition-stabilized (IS) network. The behavior of the IS network is particularly worth taking the time to understand because there is evidence that regions of both the hippocampus and the cortex could operate in the IS regime.

The IS network is a feedback-dominated network in which self-excitation is strong enough to destabilize excitatory firing rates in the absence of feedback inhibition. However, feedback inhibition is also very strong, in fact dominant enough to clamp the excitatory firing at what is an otherwise unstable fixed point of the dynamics ^17^. In IS circuits, the strong feedback inhibition is to similarly tuned neurons—to the same cells they receive excitation from—unlike most point attractor models in which the dominant inhibitory effect is cross-inhibition between differently tuned cells to enhance selectivity.

**Figure 3.**
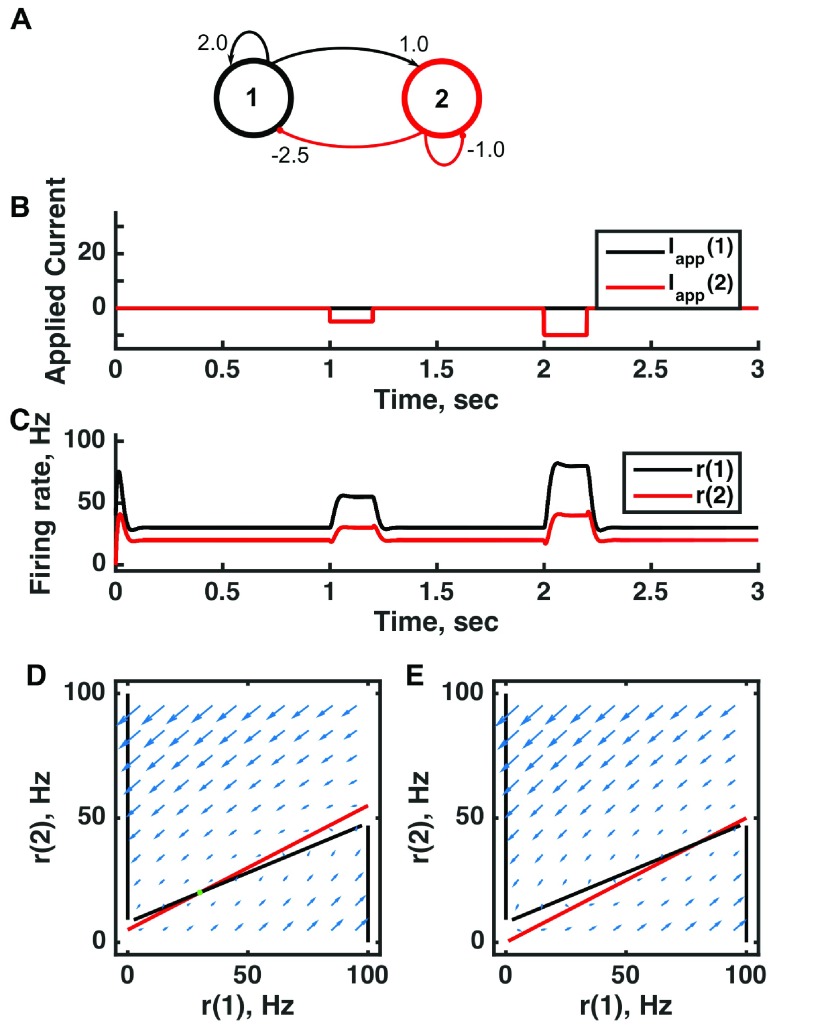
The “paradoxical” shift of a single point attractor in the inhibition-stabilized regime in a threshold-linear two-unit circuit. ( **A**) Diagram of the model circuit. Arrows indicate excitatory connections, and balls indicate inhibitory connections between units. Architecture is identical to that in Figure 1. ( **B**) Applied current as a function of time. Two different sized inhibitory pulses of current are applied to unit 2. ( **C**) Firing rate as a function of time in the coupled network. During each current step, a new attractor is produced, but following current offset the original activity state is reached. When inhibition is applied to unit 2, the rates of both unit 1 and unit 2 increase as a result of the ensuing net within-circuit increase in excitation to both units. The “paradox” lies in that external inhibition to unit 2 results in an increase in its rate (due to internal raised excitation) and, even more counter-intuitively, unit 1 stabilizes at a higher firing rate in the presence of greater inhibitory input from unit 2. ( **D**) Any particular combination of the firing rates of the two units (x-axis is rate of unit 1, y-axis is rate of unit 2) determines the way those firing rates change in time (arrows). Starting from any pair of firing rates, any trajectory following arrows terminates at the point of intersection of the two lines. Red line: nullcline for unit 2—the value of r(2) at which dr(2)/dt = 0 (its fixed point) given a value of r(1). Since unit 1 excites unit 2, the fixed point for r(2) increases with r(1). Black line: nullcline for unit 1—the value of r(1) at which dr(1)/dt = 0 (its fixed point) given a value of r(2). Since unit 2 inhibits unit 1, at high enough r(2) only r(1) = 0 is possible and at low r(2) only r(1) = 100 is possible. The line joining the minimum and maximum values of r(1) would normally be of unstable fixed points, but in this system it is stabilized. The crossing point of the nullclines is the fixed point of the whole system. The fixed point is stable (so is an attractor state) because arrows converge on the fixed point. ( **E**) As in ( **D**) but the solution during the second pulse of applied current. The inhibitory applied current shifts the nullcline for r(2) down, and the result is that the fixed point of the system moves to higher values of both r(1) and r(2). For parameters, see supporting Matlab code, “dynamics_two_units.m”.

An intriguing property of the IS network is that a decrease in external excitatory input to inhibitory cells causes their steady-state firing rate to increase ^17^. The initial transient decrease in firing of the inhibitory cells causes a strong increase in firing rate of the excitatory cells. The feedback loop is strong enough that the net effect on inhibitory cells, following the ensuing increase in their excitatory input, is an increase in firing rate ( Figure 3). In the final state, with decreased external excitatory input to inhibitory cells, both inhibitory and excitatory cells have higher firing rate. The effect can be called paradoxical because in the final state the excitatory cells fire at a higher rate while receiving more inhibitory input than before.

Evidence for neural circuit operation in the IS regime was first provided by a combination of modeling and data analysis for the hippocampus during theta oscillations, based on the relative phase relationship of oscillatory activity from excitatory and inhibitory cells ^17^. More recently, strong evidence for operation in this regime has been provided for the primary visual cortex as an explanation of how two stimuli can switch from producing supralinear to sublinear summation as their contrast increases and the IS regime is reached ^18, 19^.

### Attractor-state itinerancy

The term itinerancy is used if a system switches rapidly between distinguishable patterns of activity that last significantly longer than the switching time. Switches can occur by noise-driven fluctuations in a circuit with many stable point attractors ^20^ or via biological processes such as synaptic depression or firing rate adaptation—which operate more slowly than changes in firing rates of cells—between quasistable attractor states ^21^. A system with just two quasistable states can give rise to a relaxation oscillator by the latter process ^1, 22, 23^, whereas a system with many quasistable states can give rise to “chaotic itinerancy” ^24, 25^.

Itinerancy through quasistable states can subserve sequence memory, with distinct states reached in response to both the number of stimuli and the types of stimuli in a sequence ^21^. Noise-induced itinerancy through point attractor states can also serve as the neural basis of a sampling framework for Bayesian computation ^26^ and can lead to optimal decision making if certain biological constraints must be met by the system ^27^.

Perhaps the most compelling evidence for attractor state itinerancy is during bistable percepts ^28– 32^, when the switching from one percept to the other and back arises in the presence of a constant, ambiguous stimulus such as the Necker cube ^33^. Models of the phenomenon suggest a neural circuit possessing two attractor states with transitions between them produced by a combination of synaptic depression and noise-driven fluctuations ^34– 36^.

It is worth noting that experimental identification of such attractor state itinerancy may require non-standard analyses of neural spike trains. The standard practice of averaging across trials after their alignment to stimulus onset would fail to reveal such inherent dynamics because the timing of transitions varies across trials and indeed the initial state may differ from trial to trial. Thus, across-trial averaging would reveal simply a blurred, approximately constant rate dependent on the average response during the two distinct percepts. Therefore, it is essential to use single-trial methods of analysis where possible if one hopes to uncover such underlying dynamics. The use of averaging across trials has been a necessity when analyzing spike trains *in vivo* because of the apparent randomness and limited amount of information contained within the spike times of any one neuron in any one trial. However, methods such as hidden Markov modeling (HMM) ^37– 39^ allow one to treat each trial independently, especially if one has access to spike trains from many simultaneously recorded cells with correlated activity. HMM produces an analysis that assumes discrete states of activity with transitions between these activity states that vary in timing from trial to trial. Analysis of neural spike trains *in vivo* in multiple cortical areas by HMM and other methods has produced strong evidence for state transitions during cognitive processes, including motor preparation ^37, 40^, taste processing ^39^, and perceptual decision making ^41, 42^.

### Addressing the unrealistic firing rates in multistable models

The multistability necessary for memory is typically achieved in models via a subset of neurons switching from a low firing rate where the feedback activity is insufficient to generate a sustained response to a high firing rate state reinforced by recurrent excitatory feedback that is limited in rate only by saturation of an intrinsic or synaptic process. Unless the saturating process has a slow time constant, such active states maintained by recurrent feedback typically have much higher firing rates than those observed *in vivo*—indeed, in simple models, once excitatory feedback is increased enough to engender stable persistent activity in the absence of input, that activity can be at the neuron’s maximal firing rate, on the order of 100 Hz (as in Figure 2). Such rates are incompatible with the smaller changes of activity—often no more than 10 Hz between pre-stimulus and post-stimulus levels—during memory tasks *in vivo* ^43, 44^, calling into question the validity of these recurrent excitatory models.

**Figure 4.**
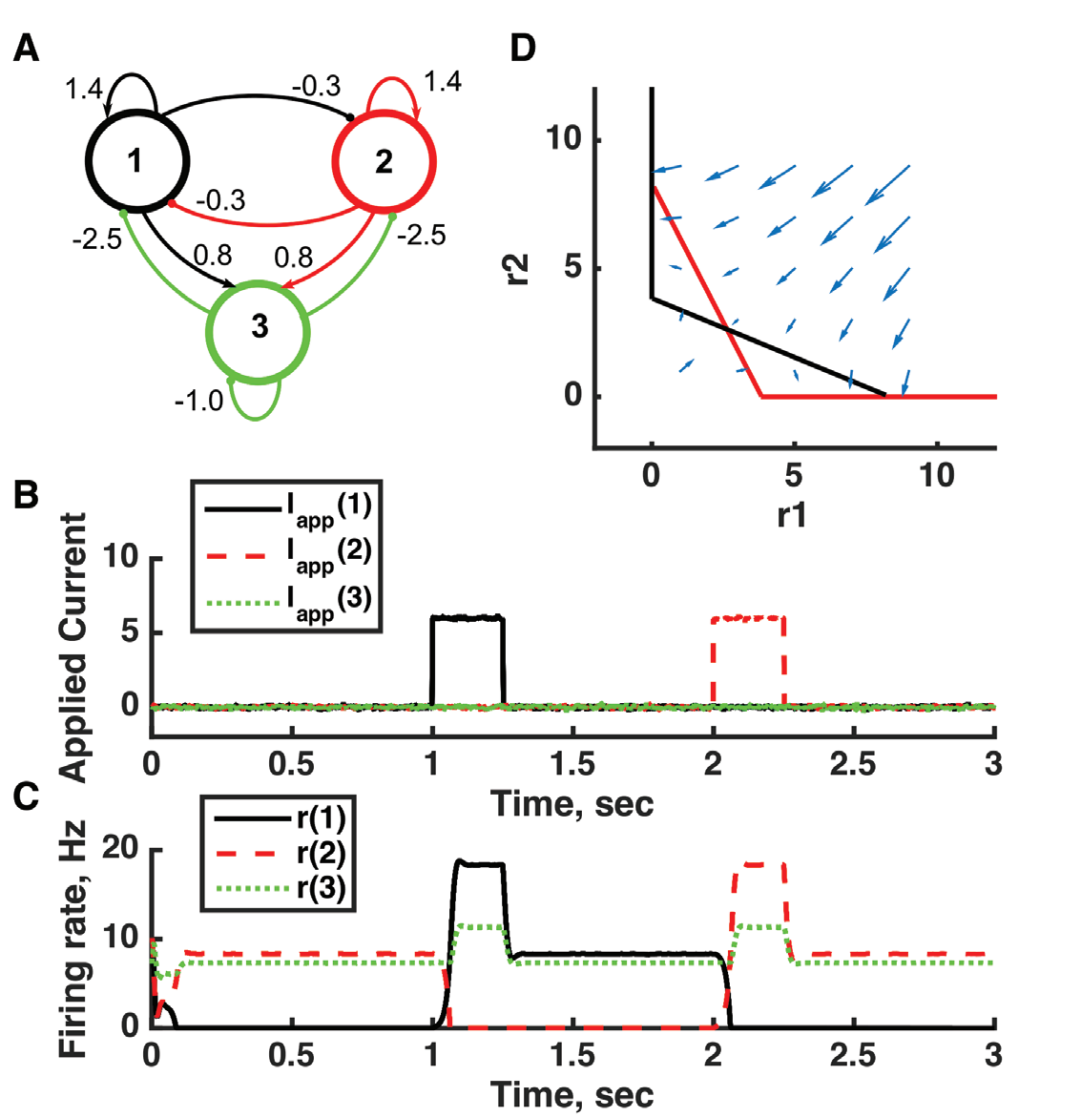
Bistability with low stable firing rates in the inhibition-stabilized threshold-linear three-unit circuit. ( **A**) Diagram of the model circuit. Arrows indicate excitatory connections, and balls indicate inhibitory connections between units. A third unit is added to the architecture of Figure 3. ( **B**) Applied current as a function of time. The first current pulse is applied to unit 1, and the second is applied to unit 3. A small amount of noise is added to the current to prevent the system resting at an unstable symmetric state, toward which it is otherwise drawn. ( **C**) Firing rate as a function of time in the coupled network. The current steps switch activity between stable states in which no neuron’s activity is greater than 10 Hz—note the difference in rates from the “traditional” bistability of Figure 2. ( **D**) Any particular combination of the firing rates of the three units (x-axis is rate of unit 1, y-axis is rate of unit 3) determines the way those firing rates change in time (arrows). Only a plane out of the full three-dimensional space of arrows is shown—the plane corresponding to dr(3)/dt = 0. Starting from any pair of firing rates, any trajectory following arrows terminates at the point of intersection of the two lines. Red line: nullcline for unit 2—the value of r(2) at which dr(2)/dt = 0 (its fixed point) given a value of r(1). Black line: nullcline for unit 1—the value of r(1) at which dr(1)/dt = 0 (its fixed point) given a value of r(2). Since unit 2 inhibits neuron 1, the fixed point for r(1) decreases with r(2). The crossing points of the nullclines are fixed points of the whole system. The asymmetric fixed points are stable (so are attractor states) because arrows converge on them, whereas the intervening symmetric fixed point is unstable. For parameters, see supporting Matlab code, “dynamics_three_units.m”.

This issue can be resolved by several different modeling assumptions ^45^. If recurrent feedback current is mediated primarily through N-methyl-D-aspartate (NMDA) receptors and they are allowed a 100 ms time constant (though 50 ms may be more reasonable at *in vivo* temperatures for mammals), then the high firing rate state can be limited to 20 to 30 Hz ^46, 47^. Furthermore, if a slow time constant for synaptic facilitation of 7 seconds is used, then firing rates in the active state can be reduced to levels below 10 Hz ^48^. A final possibility is that the network contains subgroups of excitatory and inhibitory cells operating in the IS regime ( Figure 4) in which active subgroups can, in principle, maintain stable activity at arbitrary low rates while suppressing the activity of other subgroups via cross-inhibition ^49– 51^. In this regime, a system of point attractor states is compatible with the low firing rates of persistently active neurons observed *in vivo*.

A problem related to the one above is that most models of bistability produce spike patterns in the high-activity state that are much more regular than those observed *in vivo*. Systems that transition between various stable states will cause spike trains to be less regular because of the contribution of rate variation. Lower firing rates will likely also help with this problem because most model neurons produce irregular spike trains when operating below threshold in a fluctuation-driven regime at low firing rates. However, spike statistics such as the coefficient of variation (CV) of interspike intervals have not been analyzed to date in models of the IS regime.

## Marginal states (line attractors or continuous attractors)

**Figure 5.**
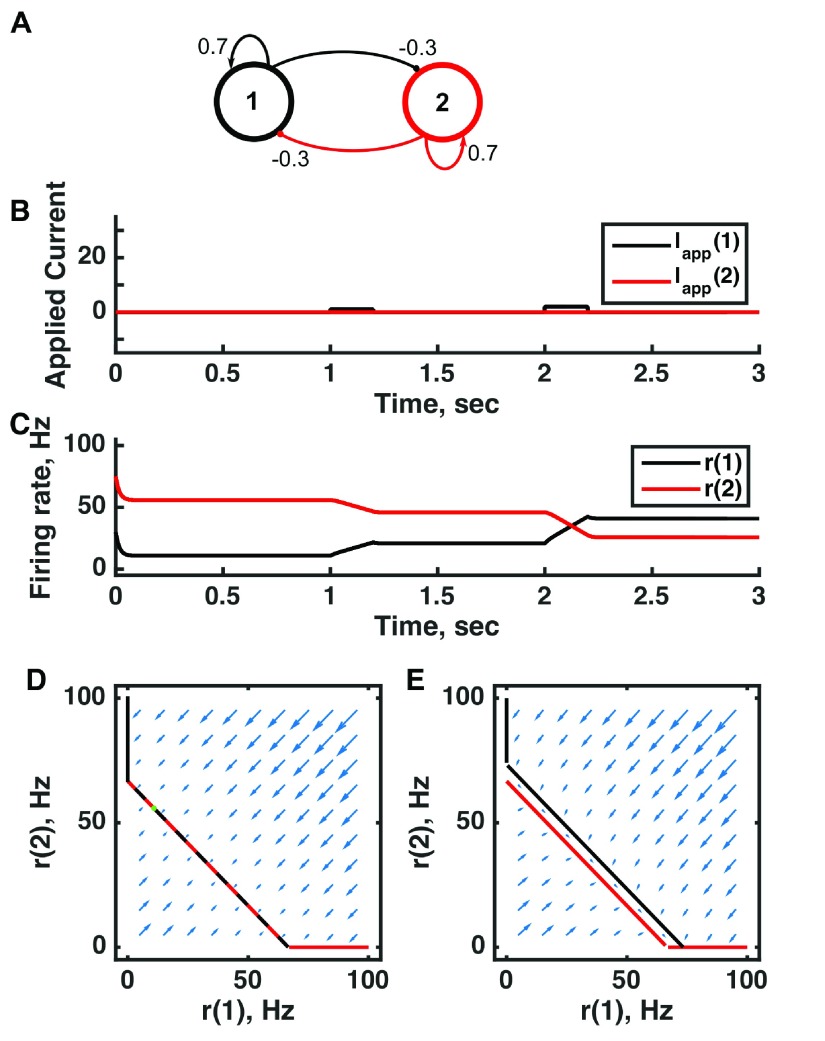
A marginal state or continuous/line attractor is produced by careful tuning of the connection strengths in a threshold-linear two-unit circuit. ( **A**) Diagram of the model circuit. Arrows indicate excitatory connections, and balls indicate inhibitory connections between units. The architecture is identical to that of Figure 2. ( **B**) Applied current as a function of time. Two very small pulses of current are applied to unit 1. ( **C**) Firing rate as a function of time in the coupled network. During each current step, the firing rate of unit 1 increases linearly because of the applied current. Inhibitory feedback to unit 2 causes a linear decrease in its firing rate. Upon stimulus offset, the firing rate reached is maintained. ( **D**) Any particular combination of the firing rates of the two units (x-axis is rate of unit 1, y-axis is rate of unit 2) determines the way those firing rates change in time (arrows). Starting from any pair of firing rates, any trajectory following arrows terminates at a point where the two lines overlap each other. Red line: nullcline for unit 2—the value of r(2) at which dr(2)/dt = 0 (its fixed point) given a value of r(1). Black line: nullcline for unit 1—the value of r(1) at which dr(1)/dt = 0 (its fixed point) given a value of r(2). Since the two units inhibit each other, the overlapping lines have negative gradient. ( **E**) A small applied current to unit 1 shifts its nullcline to the right slightly. Now the only fixed point is where the two lines intersect at r(2) = 0, but in the region between the two parallel nullclines, the rate of change is small (note the small arrows parallel to the lines) so firing rates change gradually. For parameters, see supporting Matlab code, “dynamics_two_units.m”.

If a dynamical system possesses a continuous range of points (a line) that variables of the system approach, then the attractor state has “marginal stability”; if the activity is perturbed away from the line, then it recovers toward the line, but deviations along the line can accumulate over time ^52^. In neural circuit models, marginal states either depend on an underlying symmetry (for example, translation in space when considering memory for position via a ring attractor) ^53– 56^ or require other fine-tuning of parameters ^57– 60^. When neural activity enters a marginal state, the whole system can be described by a reduced number of variables, such as the position along the line of a line attractor ^61^. Systems with marginal states are able to encode and store the values of continuous quantities ^53, 54, 60^, integrate information over time perfectly ^55, 57, 62^ ( Figure 5), combine prior information with sensory input in a Bayesian manner ^63^, and in general achieve optimal computational performance ^64^. In practice, a system with many point attractor states that are close to each other—that is, total circuit activity differs little between states—can appear like a line attractor, performing integration ^8^ yet with the benefit of greater robustness and stability ^65^.

Predicted experimental signatures of marginal states include drift of neural activity as noise accumulates in the manner of a random walk—leading to variance increasing linearly with time ^54^—in the presence of a constant stimulus; perfect temporal integration of inputs ^55, 57^; and correlations within single neural spike trains that decay linearly over time ^52, 66^. These have acquired some degree of experimental support ^67– 69^.

## Oscillating systems (cyclic attractors or limit cycles)

Observations of oscillations are widespread throughout the brain ^70, 71^—indeed, the earliest human extracranial recordings revealed oscillations in electrical potential ^72^. In some cases, the presence of oscillations is inferred from a peak in the power spectrum at a particular frequency ^73, 74^—such a peak could arise from attractor state itinerancy, chaos, or heteroclinic orbits (see below) in the absence of a true oscillator. Yet, given the overwhelming abundance of evidence, there is no doubt as to the existence of oscillations in neural circuits—something still in question for other dynamical frameworks discussed here. Therefore, current research focuses on elucidating the role, if any, of oscillations in diverse mental processes ^75, 76^.

**Figure 6.**
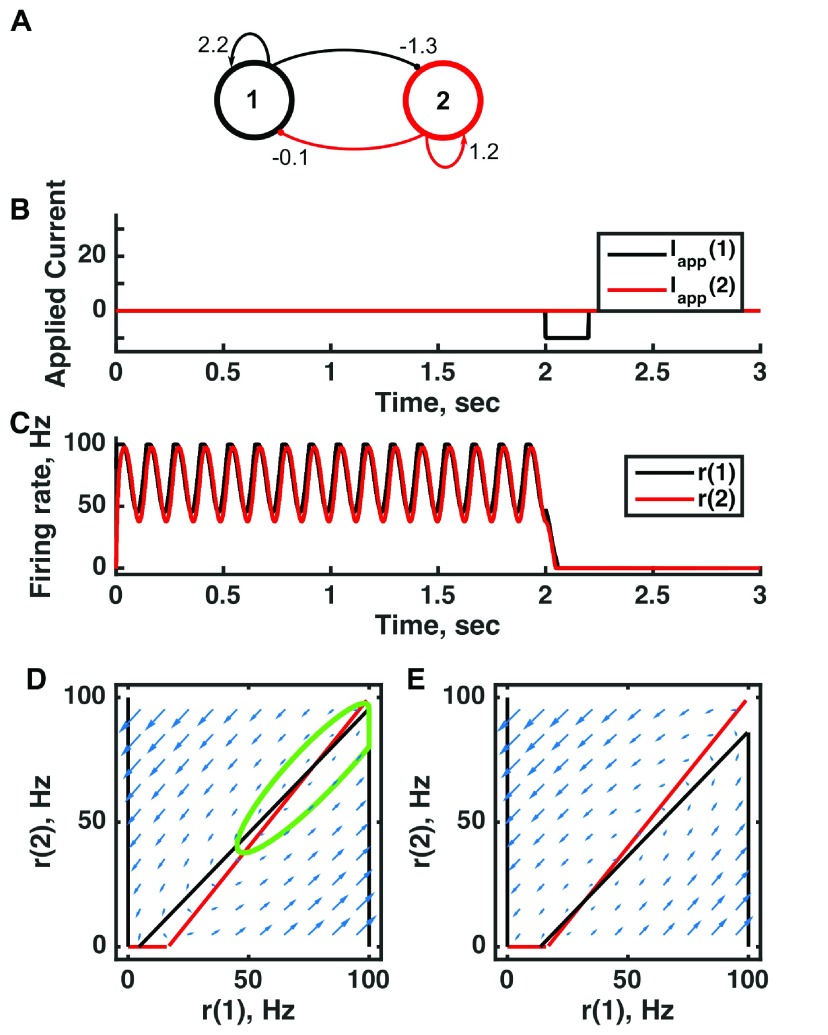
An oscillator produced by directed excitatory and inhibitory connections in a bistable threshold-linear two-unit circuit. ( **A**) Diagram of the model circuit. Arrows indicate excitatory connections, and balls indicate inhibitory connections between units. The architecture is identical to that of Figure 1 and Figure 3. ( **B**) Applied current as a function of time. An inhibitory pulse of current is applied to unit 1. ( **C**) Firing rate as a function of time in the coupled network. Oscillations are switched off by the inhibition to unit 1, so the system has two stable attractors: one a limit cycle, the other a point attractor. ( **D**) Any particular combination of the firing rates of the two units (x-axis is rate of unit 1, y-axis is rate of unit 2) determines the way those firing rates change in time (arrows). Depending on the starting point, a trajectory following the arrows will fall on the limit cycle (the green closed orbit, which represents the coordinated variation of firing rate with time during the oscillations) or will reach the fixed point at the origin. Red line: nullcline for unit 2—the value of r(2) at which dr(2)/dt = 0 (its fixed point) given a value of r(1). Since unit 1 excites unit 2, the fixed point for r(2) increases with r(1). Black line: nullcline for unit 1—the value of r(1) at which dr(1)/dt = 0 (its fixed point) given a value of r(2). Crossing points of the nullclines are the fixed points of the whole system, but the one within the limit cycle is unstable, as is the one with r(2) = 0 but r(1) > 0. ( **E**) As in ( **D**) but the solution during the inhibitory pulse of applied current. The inhibitory current shifts the nullcline for r(1) down, and the result is that all trajectories terminate at the origin. For parameters, see supporting Matlab code, “dynamics_two_units.m”.

**Figure 7.**
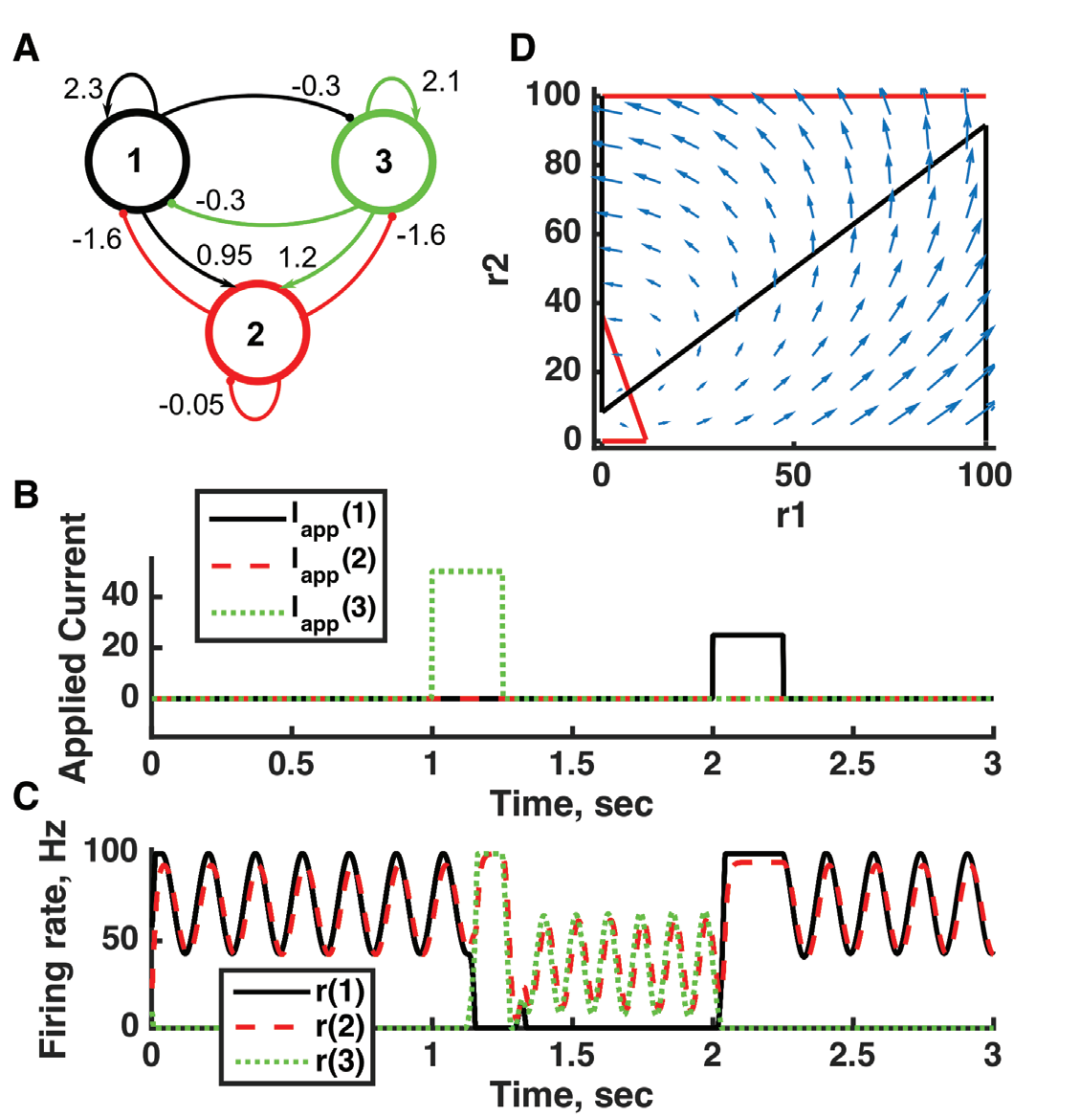
Switching between two distinct oscillators in a bistable threshold-linear three-unit circuit. ( **A**) Diagram of the model circuit. Arrows indicate excitatory connections, and balls indicate inhibitory connections between units. Architecture is identical to that of Figure 4. ( **B**) Applied current as a function of time. The first current pulse is applied to unit 3, and the second is applied to unit 1. ( **C**) Firing rate as a function of time in the coupled network. The current steps switch activity between two stable states with different frequencies of oscillation. ( **D**) Any particular combination of the firing rates of the three units (x-axis is rate of unit 1, y-axis is rate of unit 3) determines the way those firing rates change in time (arrows). Only a plane out of the full three-dimensional space of arrows is shown: the plane corresponding to dr(3)/dt = 0. Red line: nullcline for unit 2—the value of r(2) at which dr(2)/dt = 0 (its fixed point) given a value of r(1). Black line: nullcline for unit 1—the value of r(1) at which dr(1)/dt = 0 (its fixed point) given a value of r(2). The crossing points of the nullclines are fixed points of the whole system, none of which is stable in this example. For parameters, see supporting Matlab code, “dynamics_three_units.m”.

Spiking neurons themselves can be oscillators, so it should not seem surprising that neural circuits can also oscillate. Circuit oscillations can arise from the intrinsic oscillations of constituent neurons, or from the circuit connectivity ( Figure 6), or from a combination of the two. In general, any system with fast positive feedback and slower negative feedback is liable to oscillate ( Figure 7). Moreover, in any nonlinear dynamical system, a change in the inputs leads to a change in amplitude and frequency of ongoing oscillations, so correlations between oscillatory power or frequency and task condition are inevitable. Therefore, observed correlations between oscillatory power or synchrony and behavior or cognitive process—whether related to attention ^77^, arousal ^78^, memory load ^79^, or sleep state ^80– 82^—may indicate a causal dependence in one direction (oscillations cause the process) or the other (particular processes cause oscillations as an epiphenomenon). The difficulty of distinguishing the two arises because experiments aimed at altering an oscillation inevitably alter other properties of the dynamical system necessary for the cognitive process.

The importance of oscillations is perhaps undisputed only in the case of motor systems that produce a repeating, periodic output ^83^ or in the sensations of whisking and olfaction that are directly related to such motor rhythms. Perhaps the most accepted roles for information processing by oscillations are within hippocampal place cells, whose phase of firing with respect to the ongoing 7 to 10 Hz theta oscillation contains substantial information ^84– 87^.

Oscillating systems contain limit cycles and so appear as closed loops in a plot of one variable against another ( Figure 6C). Since activity is attracted to the limit cycle—which is a line embedded within a higher-dimensional space—oscillating systems have some similarities to line attractors. In particular, small perturbations can be accumulated in the phase of the oscillation (along the line of the limit cycle), so, as with line attractors, noise accumulates as a random walk in one particular direction. Moreover, the phase of an oscillator retains a memory of perturbations, so oscillators can also be integrators, albeit only up to an offset of one cycle and with the need of an unperturbed oscillator for comparison.

## Chaotic systems (strange attractors)

A high-dimensional neural system—as arises if the activity of each neuron can vary with little correlation with the activity of other neurons—with a balance between excitatory and inhibitory random connections becomes chaotic if connections are strong enough ^88– 90^. An early model of eye-blink conditioning in the cerebellum (the part of the mammalian brain with the highest density of cells and connections) used a chaotic circuit to encode and reproduce timing information ^91, 92^. 

**Figure 8.**
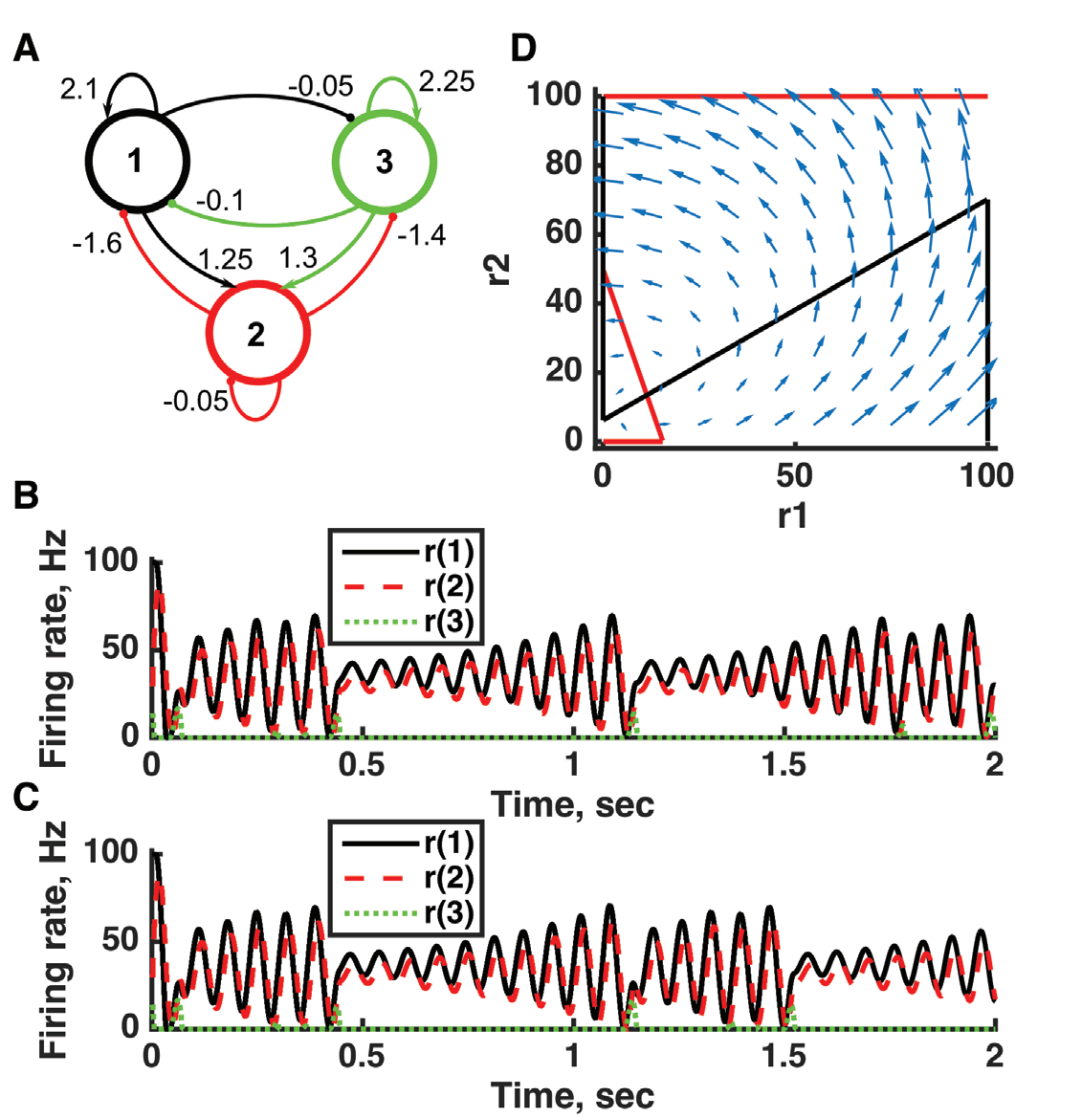
Chaotic activity in a threshold-linear three-unit circuit. ( **A**) Diagram of the model circuit. Arrows indicate excitatory connections, and balls indicate inhibitory connections between units. Architecture is identical to that of Figure 4 and Figure 7. ( **B, C**) Firing rate as a function of time in the coupled network, from two imperceptibly different initial conditions. No applied current is present. The miniscule difference in initial conditions is amplified over time, so, for example, unit 3 produces a small burst of activity after 1.5 s in ( **D**), but that burst is absent from ( **C**). ( **D**) Any particular combination of the firing rates of the three units (x-axis is rate of unit 1, y-axis is rate of unit 3) determines the way those firing rates change in time (arrows). Only a plane out of the full three-dimensional space of arrows is shown: the plane corresponding to dr(2)/dt = 0. Red line: nullcline for unit 2—the value of r(2) at which dr(2)/dt = 0 (its fixed point) given a value of r(1). Black line: nullcline for unit 1—the value of r(1) at which dr(1)/dt = 0 (its fixed point) given a value of r(2). The crossing points of the nullclines are unstable fixed points of the whole system. For parameters, see supporting Matlab code, “dynamics_three_units.m”.

The smallest change in the initial conditions of a chaotic system leads to an indeterminate change in response, which can pose a serious problem for information processing and memory ( Figure 8). Yet a system operating near or at the “edge of chaos” can be computationally efficient ^93^ and become reliably entrained to inputs while responding more rapidly than ordered systems ^89, 94^. Moreover, certain learning rules for changing of connection strengths between neurons in a chaotic system can allow the encoding of almost any spatiotemporal input pattern ^4, 95^, the switching between multiple patterns ^4^, and the encoding and processing of many rule-based tasks ^6, 8^. Thus, chaotic systems appear to be highly flexible and trainable.

Observed signatures of chaos include the apparent randomness and variability of spike trains, especially during spontaneous activity in the absence of stimuli, and the initial drop of such variability upon stimulus presentation ^94, 96^.

Chaos can arise in systems with quasistable attractor states as an itinerancy between the states in an order possessing no pattern ^97, 98^ or in an oscillating system (typically as unpredictable jumps between different types of oscillation). Heteroclinic sequences (see below) can also be chaotic ^99^. Addition of a small amount of intrinsic noise to chaotic systems causes the divergence in activity observed without noise upon small changes in initial conditions to occur on separate trials with identical initial conditions.

## Heteroclinics

A common type of fixed point in a system with many variables (meaning it is high-dimensional) is a saddle point. Saddle points are so-named because, like the saddle on a horse or the saddle on a ridge, there are directions where the natural tendency is to approach the fixed point (moving down from a higher point on the ridge) and other directions where the natural tendency is to move away from the fixed point (down to the valley below). If the state of a dynamical system can move toward one such saddle point and then move away from it to another one, and so on, the trajectory is called a heteroclinic sequence.

**Figure 9.**
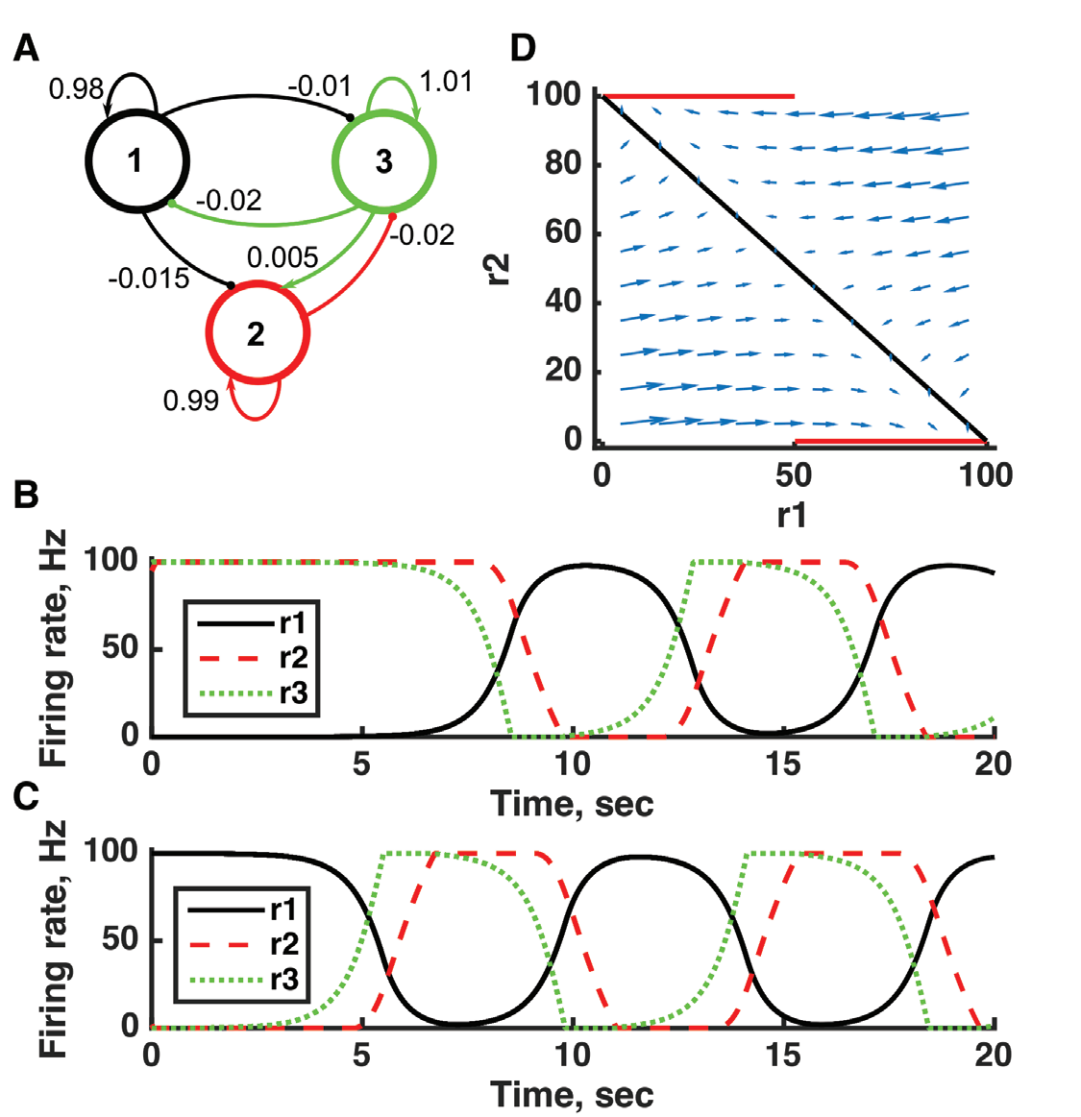
Heteroclinic orbits in a threshold-linear three-unit circuit. ( **A**) Diagram of the model circuit. Arrows indicate excitatory connections, and balls indicate inhibitory connections between units. ( **B, C**) Firing rate as a function of time in the coupled network from different initial conditions (near the fixed points) with no applied current. Activity is initially slow to move away from the vicinity of the fixed point (along its unstable direction) but after a cycle returns to the vicinity of the same fixed point (along its stable direction). ( **D**) Any particular combination of the firing rates of the three units (x-axis is rate of unit 1, y-axis is rate of unit 3) determines the way those firing rates change in time (arrows). Only a plane out of the full three-dimensional space of arrows is shown: the plane corresponding to dr(2)/dt = 0. Red line: nullcline for unit 2—the value of r(2) at which dr(2)/dt = 0 (its fixed point) given a value of r(1). Black line: nullcline for unit 1—the value of r(1) at which dr(1)/dt = 0 (its fixed point) given a value of r(2). The crossing points of the nullclines are fixed points of the whole system, in this case at r(1) = 100, r(2) = 0, and r(3) = 0 and at r(1) = 0, r(2) = 100, and r(3) = 100. The fixed points are saddle points in that firing rates can either approach the fixed point or move away from it, depending on the precise set of rates. For parameters, see supporting Matlab code, “dynamics_three_units.m”.

Heteroclinic sequences have similarities to systems with attractor-state itinerancy and a type of oscillator that switches between states that appear stable on a short timescale, called a relaxation oscillator. All three systems have states toward which the system is drawn but at which the system does not remain. In a heteroclinic sequence, activity can be funneled toward each saddle point as if it were an attractor state but, once in the vicinity of the saddle point, will find the direction of instability and move away ( Figure 9). In the absence of noise, the duration in a “state” (the vicinity of a particular saddle point) depends on how close to the fixed point the system gets and therefore may vary with initial conditions. Interestingly, a small amount of noise can make these state durations more regular ^100^.

Models of heteroclinic sequences have been proposed as a basis for memory ^101, 102^ and decision making ^103, 104^. According to some calculations, a randomly connected neural circuit would contain a suitable number of heteroclinic trajectories for information processing ^105^. In the high-dimensional space of neural activity, most random fixed points would have some directions along which they attract neural activity and other directions along which they repel it, meaning that they would be the saddle points necessary for producing heteroclinic sequences. However, no unique predictions of cognitive processing via heteroclinic sequences have been linked to empirical data to date.

## Criticality

Many scientists argue that the brain is in a critical state, in fact exhibiting self-organized criticality, so should be studied as such. Criticality is a measured state of a system rather than a dynamical model. A critical system is characterized by a number of properties, including the following: power law decays of the durations and sizes of features such as neural avalanches ^106^; relationships between the exponents of these different power laws; a scaling of the time dependence of these features when binned by size onto a universal curve ^107^; and correlations of fluctuations that extend across the system. All of these features arise from a particular distribution of the number of possible states binned according to their probability of occurrence—a distribution that, in principle, can be enumerated ^108^—that conspire to remove any stereotypical size of the systems fluctuations. Critical systems have been argued to be optimal at information processing ^93, 109^.

It is likely that most of the above dynamical systems under appropriate conditions could produce critical behavior ^110^—although the term criticality is sometimes used for systems operating at the edge of chaos ^93^ where critical properties can arise in random networks ^111^. Criticality appears to be more strongly dependent on the modular or hierarchical structure of network connectivity ^112– 114^ than the within-module dynamics.

Characteristics of criticality have been measured in cortical cultures ^106, 107^, intact retina ^108^, and whole-brain imaging ^115^. Observations to date suggest that much neural activity is close to being critical ^109, 116^ rather than exactly critical, leaving it open that even if a particular dynamical system cannot engender exact criticality, it is still close enough to provide an accurate description of neural activity (but see 108).

## Summary

Many different types of dynamical system have been proposed as models of neural activity, each of which can be justified by experimental evidence in some brain regions and circumstances. Different neural circuits in different parts of the brain may operate in different dynamical regimes because of connectivity differences, in particular differences in relative dominance of feedback or feedforward connections and in the relative contributions of excitatory or inhibitory neurons. Moreover, since the dynamical regime of a neural circuit depends on many factors (only one of which is the connectivity pattern), it is likely that a single circuit can change between dynamical regimes following learning or when it receives inputs or neural modulation.

Any one of these types of dynamical system (or any other not mentioned) could provide the most accurate basis for understanding a particular neurological function, another may be compatible with the observed data and more useful for explaining particular features of the neural behavior, while others may at heart be incompatible with the known circuit properties. Therefore, it behooves us all to avoid entrenchment in our favorite paradigm and to improve our understanding of the variety of dynamical systems when attempting to understand neural circuit function.

A final point worth making is that just knowing the connectome—which neurons are connected to each other—does not tell us about the operation of a neural circuit. Many of the dynamics we see arise in the same simple circuit with the same neurons (for example, Figure 1, Figure 3, and Figure 6 have identical architecture, even accounting for type of synapse, as do Figure 4, Figure 7, and Figure 8); differences in neural excitability or differences in strengths of connections can produce different functionality within a single architecture. Conversely, distinct connectivity patterns can give rise to the same function. Rather, observation of the coordinated activity of many neurons during mental processing is the route to understanding the remarkable abilities of our brains.

## Supplementary material

dynamics_two_units.

.

dynamics_three_units.
